# The Clinical Society

**Published:** 1896-12-19

**Authors:** 


### Medical Progress and Hospital Clinics.

[*The Editor will be glad to receive offers of co-operation and contributions from members of the profession. All letter should be addressed to* The Editor, at the Office, 28 & 29, Southampton Street, Strand, London, W.C.]

### THE CLINICAL SOCIETY.

The subject of renal tension, which was recently discussed by Mr. Reginald Harrison at another society, was brought forward again by Dr. David Newman, of Glasgow, who related several cases illustrating increased vascular tension of the kidney causing hæmaturia and renal pain, with and without albuminuria, which were cured by operation.

That very obscure and very unsatisfactory subject of tumour of the cerebellum was certainly brightened up by the relation by Mr. Parkin of a case in which he had removed a cerebellar tumour two and a half years ago, and in which, so far, there had been no return of symptoms. Before the operation not only had the symptoms been very pronounced, but the patient (a child) had been so ill as to appear in imminent danger of death. Gradually, however, after the operation, all these indications of disease had passed away, so that now the child could walk and talk, and there was very little to show what a serious condition had existed. The President, in commenting on the communication, said that cases like this were like good wine, and improved by age, for there could be no doubt that the importance of what Mr. Parkin had told them was greatly increased by the fact that there had been no recurrence, notwithstanding that two years had elapsed since the operation was performed.

The uncertainties and surprises which are so frequent in abdominal surgery were well illustrated in a case related by Dr. H. D. Rolleston and Mr. Marmaduke Sheild, in which excision of the cæcum was performed. The patient was a woman aged twenty-nine, who was admitted to St. George's Hospital on July 27th. She had had a lump in the right side of the abdomen ever since the birth of her last child in February. The tumour, which was hard and the size of an orange, moved about freely and caused pain, but there was no obstruction and no blood or slime in the evacuations. There was no evidence of renal pain. On August 22nd Mr. Sheild exposed the tumour by a curved incision. There was a long meso-colon. The mass was found to be in the cæcum, which on being brought forward burst, and a large cancerous mass was exposed—in fact, became at once everted into the peritoneal cavity. It was found that the glands were extensively infiltrated. The cæcum, together with the growth, was excised, and the glands also were removed, a proceeding which involved the loss of a large amount of mesentery, the pedicle having to be tied quite close to the spine. A portion of the colon was invaginated so as to equalise the parts to be united, and then the ilium and colon were approximated, end to end, and sewn on the finger without the aid of any apparatus. The parts were flushed and dusted with iodoform, and iodoform gauze was packed around, the ends being brought out so as to form a drain. About four and a half inches of the cæcum and ascending colon were removed, the growth extending to and around tho ilio-cæcal valve. In structure it was columnar-celled carcinoma. The gauze was removed on the second day, after which the sinus was regularly filled with iodoform emulsion. Twelve days after the operation a more or less fæcal discharge occurred from the wound, which however soon ceased; and on October 12th, when the patient got up, the wound had practically healed. There was, however, still, in all probability, a slight communication with the bowel, this being due to the large removal of the mesentery which was found necessary in consequence of the extensive infiltration of the glands and the resulting interference with the nutrition of the parts involved in the operation. In regard to the diagnosis it was important to note the very few symptoms which existed pointing to any disturbance of the gastro-intestinal tract, which was curious, considering that the growth surrounded the ilio-cæcal valve. The prominence of the tumour and the ease with which it could be felt were worth noting. The hardness was against floating kidney, and, moreover, the right kidney could be felt in its position. The tumour was clearly unconnected with the liver. In the discussion a member related a case in which he implanted the cut end of the ilium into the side of the colon, the divided end of the latter being closed by inversion. He thus in some degree reproduced an ilio-cæcal valve.

Mr. Walsham related two cases of cancer of the cæcum, in one of which he had performed excision, producing lateral approximation of the ilium and colon by means of Senn's plates, and in the other had united the ilium to the colon by a Murphy's button, without removing the growth. In reply Mr. Sheild said that he did not consider end to end junction the best method in such cases. If all could be prepared beforehand there could be no doubt that lateral approximation by means of Senn's plates was the best procedure, but in this case he had to deal with the matter immediately.

